# The effects of potentially traumatic events on the recovery from pre‐existing anxiety and depression symptomatology and the risk of PTSD

**DOI:** 10.1111/pcn.13725

**Published:** 2024-08-20

**Authors:** Peter G. van der Velden, Carlo Contino, Lonneke Lenferink, Marcel Das, Lutz Wittmann

**Affiliations:** ^1^ Tranzo, Tilburg School of Social and Behavioral Sciences, Tilburg University Tilburg The Netherlands; ^2^ Centerdata Tilburg The Netherlands; ^3^ Fonds Slachtofferhulp The Hague The Netherlands; ^4^ University of Twente Enschede The Netherlands; ^5^ International Psychoanalytic University Berlin Berlin Germany

**Keywords:** anxiety, depression, logistic models, post‐traumatic stress disorder, prospective studies

## Abstract

**Aim:**

The extent to which recent potentially traumatic events (PTEs) hinder the recovery from pre‐existing mental health problems is largely unknown. The same applies to the extent to which non‐recovery from pre‐existing mental health problems increases the risk of posttraumatic stress disorder (PTSD). The aim of the present study is to gain insight in these effects.

**Methods:**

Data were extracted from six annual surveys of the Dutch population‐based Victims in Modern Society (VICTIMS) study. Of the adult respondents who participated in two subsequent surveys (labeled T1 and T2, *n =* 6942), those with severe anxiety and depression symptoms (ADS) at T1 (*n* = 487) were selected. We distinguished respondents exposed to PTEs (PTE‐group, *n* = 162) and not exposed to PTEs (comparison group, *n* = 325) between T1 and T2. We applied five indicators of recovery [based on the Reliable Change Index (RCI), degrees of symptom reduction, and the cut‐off score at T2]. Differences in the recovery from ADS and probable PTSD at T2 were examined using multivariate logistic regression.

**Results:**

The PTE group less often recovered from severe ADS between T1 and T2 than the comparison group according to all five indicators of recovery, while controlling for 11 different variables (0.40 ≤ adjusted OR's ≤ 0.66). Those in the PTE group who did not recover, considerably more often suffered from probable PTSD at T2 (63%–82%) than those who did recover (0%–29%; 8.96 ≤ adjusted OR ≤ 26.33).

**Conclusion:**

Recent potentially traumatic events hinder the recovery from pre‐existing anxiety and depression symptomatology and thereby increase the risk of probable PTSD.

## Introduction

Almost all studies on the effects of potentially traumatic events (PTEs) on the mental health of adults (civilians) were, for obvious reasons, initiated after the events occurred. They therefore lack non‐retrospective data on pre‐existing or pre‐event mental health problems such as anxiety and depression symptomatology. Retrospective collected data on pre‐event mental health problems or disorders may be sensitive to recall bias.^1^, ^2^, ^3^, ^4^

However, given the estimated prevalence of mental health problems and disorders among the general adult population^5^ we may safely assume that part of adults exposed to PTEs had pre‐existing or pre‐event mental health problems or disorders. In other words, and without neglecting the burden of suffering from post‐event mental health problems and disorders, it is unlikely that PTEs are by definition the starting point of mental health problems and disorders. For instance, a recent study among patients of motor vehicle collisions (MVC) showed that probable PTSD and depression disorder in the 30 days before the MVC, assessed during stay at the emergency department, was present among 20.5% and 6.2% of the patients, respectively.^6^ In fact, a growing number of prospective (review) studies revealed that many variables considered outcomes of exposure to PTEs were present before the PTEs.^7^, ^8^, ^9^ A recent prospective study^10^ showed that the large majority of adults exposed to PTEs with post‐event anxiety and depression symptoms or probable posttraumatic stress disorder (PTSD) already suffered from mental health problems in the 2 years before the event.

If a significant part of the adults exposed to PTEs had pre‐existing mental health problems or disorders, the question arises to what extent recent PTEs hinder the recovery from mental health problems compared to adults not exposed to PTEs with similar pre‐existing mental health problems or disorders. To the best of our knowledge, prospective comparative trauma studies to date have hardly addressed the important issue if exposure to recent PTEs leads to lower recovery rates.

The aim of the present prospective comparative population‐based two‐wave study (T1 and T2) was to gain insight into the extent to which PTEs between T1 and T2 hinder or delay recovery from pre‐existing mental health problems, in this case severe anxiety and depression symptom (ADS) levels assessed at T1. In case of a negative impact of PTE on recovery from pre‐existing severe ADS, a second aim was to test to what extent this lack of recovery increases the risk for probable PTSD at T2. Research questions were:

1. Do recent PTEs affect the recovery from pre‐existing severe ADS (RQ1)? In order to answer this question, we compared the recovery rates of severe ADS between T1 and T2 among adults exposed to PTEs between T1 and T2 (labeled PTE group) with the recovery rates of severe ADS at T1 among adults not exposed to PTEs between T1 and T2 (labeled comparison group), using five different indicators of recovery.
2. To what extent is non‐recovery from pre‐existing severe ADS associated with a higher risk of probable PTSD at T2 among adults recently exposed to PTEs (RQ2)? To answer this question, we compared the prevalence of probable PTSD at T2 among adults exposed to PTEs between T1 and T2 who did *versus* did not recover from pre‐existing severe ADS at T1 according to the five indicators of recovery.

In all analyses we controlled for 11 potential confounders such as demographics,^11^ pre‐existing financial problems,^12^ pre‐existing lack of emotional support,^13^ and pre‐existing PTSD symptoms at T1, time of participation (during COVID‐19 pandemic or not), and experiences with stressful life‐events during the study period.^14^

## Methods

### Procedures and participants

To obtain a larger number of respondents exposed to recent PTEs with severe pre‐existing ADS, we extracted and aggregated data from six annual surveys of the longitudinal Victims in Modern Society (VICTIMS)‐study (2018–2023), conducted with the Longitudinal Internet studies for the Social Sciences (LISS) panel (for all details of this study please see reference 15). This panel is based on a traditional probability sample drawn from the Dutch population register by Statistics Netherlands.^16^ The set‐up of LISS in 2007 was funded by the Dutch Research Council and is managed by Centerdata (a non‐profit research institute housed at the campus of Tilburg University, The Netherlands). Panel members receive an incentive of 15 euros per hour and complete online questionnaires every month. Members who do not have a computer and/or internet access are provided with the necessary equipment at home. All surveys were conducted in March (in April reminders were sent to non‐responders).

In accordance with the General Data Protection Regulation (GDPR), participants gave explicit digital consent for the use of the collected data for scientific and policy relevant research. The VICTIMS‐study and questionnaire was approved by an Internal Review Board of Centerdata, consisting of independent, internal and external reviewers. These reviewers were not involved in the development of the VICTIMS‐study. Since our research did not impose a certain behavior, our research did not need the approval of a Dutch Medical Ethical Testing committee according to Dutch Law. The authors assert that all procedures contributing to this work comply with the ethical standards of the relevant national and institutional committees on human experimentation and with the Helsinki Declaration of 1975, as revised in 2008.

The response rate of each survey varied between 82.4% and 87.9%. Data used for this study are available in the LISS Data Archive. This archive received the CoreTrustSeal certification, based on the World Data System (WDS) of the International Science Council and the Data Seal of Approval (DSA) catalogue and procedures. Further information about the LISS panel and free access to the data can be found at https://www.dataarchive.lissdata.nl and https://www.dataarchive.lissdata.nl/study-units/view/941 (in English).

We first selected adult respondents (18+) who participated in *two* subsequent surveys of the VICTIMS‐study, labeled T1 and T2. In case respondents participated in multiple subsequent surveys, the data of the first two subsequent surveys were used in which respondents participated. For example, in case respondents participated in the 2019, 2020 and 2021 survey, the 2019 survey was treated/labeled as T1, the 2020 survey as T2, and the 2021 survey was neglected. If another respondent participated in 2022 and 2023, the former was treated/labeled as T1, and the latter as T2. On average, 84% of the respondents of a survey participated in a subsequent survey, varying from 81.3% (of those who participated in the 2018 survey, participated in the 2019 survey) to 87.5% (of those who participated in the 2019 survey, participated in the 2020 survey). One respondent indicated by the gender self‐identification question to be intersex. Because the cell count for intersex is one and given that gender was entered as a control variable, we could not include this respondent in the analyses. This selection process resulted in 6942 unique subjects participating in two subsequent surveys.

In total, 1295 respondents were exposed to PTEs between T1 and T2 and 5647 respondents were not exposed to PTEs in this period (comparison group). We next selected respondents in the PTE group (*n* = 162) and comparison group (*n* = 325) with severe ADS (criteria see below) at T1 (*n* ^total^ = 487, 7.0%).

The COVID‐19 pandemic started in the Netherlands around March 2020 and ended around March 2022. Analyses showed no significant differences between the PTE group and comparison group in the distribution of the used two subsequent surveys during the pandemic (2020–2021, 2021–2022) and before or after the pandemic (2018–2019, 2019–2020, 2022–2023). The distribution of these two periods did not differ (during pandemic: PTE group 27.4%, and comparison group 33.3%; before or after the pandemic 62.6% and 66.7% respectively; *χ* ^2^(1) = 1.845, *P* = 0.174).

### Measures

#### Potentially traumatic events

Exposure to PTEs in the 12 months between T1 an T2 were examined at T2 by a list of 21 events with yes‐no answer categories derived from existing questionnaires on PTEs and other stressful life events.^17^, ^18^, ^19^, ^20^ Respondents could report another event they experienced in the past 12 months that was not listed, and the answer was coded into new or existing categories. The following events were defined as PTEs: (i) *physical violence*, including sexual violence/sexual abuse (not online), online sexual violence/sexual abuse, robbery, physical violence but not by own partner, and/or physical violence by own partner; (ii) *accidents*, including traffic accidents, disasters, fire, medical errors; and (iii) *serious threats*, including serious threats without the use of physical violence (not online), and/or online serious threats without use of physical violence. The VICTIMS‐study focused on adults and events in the past 12 months. PTEs such as adverse child experiences were therefore excluded.

When respondents reported two or more of these PTEs they were asked to focus on the PTE respondents found to be the most drastic or traumatic when answering event‐related questions (such as on PTSD symptoms). Other examined non‐PTE stressful life events were unexpected and expected death of a loved one or colleague, serious disease, (online) theft or fraud, contraction of a serious infection (e.g., HIV, AIDS), development of a serious physical ailment (e.g., cancer, heart attack) and burglary. In the present study we did not consider the death of a loved one or colleague as a PTE because according to the DSM5^21^ event criterion of PTSD, the event must have been violent or accidental, and we have no information about the cause of death (exposure to death was treated as a control variable, see the Analyses section below).

In addition, respondents exposed to PTEs were asked when the (most drastic) event occurred on a 8‐point scale (for the present study recoded into: 1 = 1–2 months ago, 2 = 3–4 to 5–6 months ago, 3 = 6–12 months ago) and amount of tension or stress during the (most drastic) event on a 5‐point scale (for the present study recoded into: 1 = not at all or barely to moderate, 2 = quite a bit to extremely).

#### Anxiety and depression symptoms

Anxiety and depression symptoms (ADS) during the past month were examined at T1 and T2 using the 5‐item Mental Health Index (MHI‐5).^22^, ^23^ The MHI‐5 asks respondents to rate their mental health on 6‐point Likert scales (0 = never to 5 = continuously). After recoding the three negatively formulated items, the total scores were computed and multiplied by four to arrive at a 0–100 scale (lower scores indicate higher ADS levels). We used the cut‐off score of ≤44 to calculate the prevalence of severe ADS symptom levels.^24^ Cronbach's alpha's were 0.873 and 0.875 at T1 and T2, respectively.

#### Probable PTSD

PTSD‐symptomatology during the past month according the DSM5 with respect to the (most drastic) PTE was examined at T2 with the 8‐item version of the PCL‐5.^25^ The items have a 5‐point Likert scale (0 = not at all to 4 = extremely). Higher scores reflect higher PTSD‐symptom levels. To identify adults exposed to PTEs with probable PTSD at T2 (no/yes), the cut‐off of ≥13 was applied.^26^ Cronbach's alpha was 0.962.

In the analyses (see below) we controlled for high PTSD symptoms levels at T1 following PTEs in the 12 months before T1. For high PTSD symptom levels (yes/no) the same cut‐off score of ≥13 was applied, but without the 1‐month criterion. Respondents not exposed to PTEs in the 12 months before T1 were coded as “no.”

#### Control variables

The following 11 variables were treated as control variables: (1–5) demographics at T1 (gender, age, education level, marital status, and employments status), (6) financial problems at T1, (7) use of mental health services at T1, (8) lack of emotional support in response to problems at T1, (9) time of survey (during pandemic or not), (10) high PTSD symptom levels related to PTEs in the 12 months before T1, and (11) stressful life‐events between T1 and T2Financial problems and use of mental health services at T1 were examined with the brief Problems and Help Inventarisation List (PHIL).^12^ The PHIL examines eight current problems varying from physical, mental health to financial and religious problems (1 = Yes, 2 = No). When respondents report that they have specific problems such as mental health problems, respondents are asked to indicate if they are receiving professional help for these problems such as from a general practitioner, psychiatrist, or psychologist. For the present study respondents who reported having mental health problems and reported receiving professional help for these problems, were considered as mental health services users.

Lack of emotional support in response to problems at T1 was examined using the 8‐item subscale Lack of emotional support of the Social Support List‐Discrepancy (SSL‐D).^27^, ^28^ The SSL‐D items have 4‐point Likert scales (1 = I miss this, I would like it to happen more often to 4 = It happens too often). Cronbach's Alpha was 0.885. For the present study, total scores were subtracted from the total maximum score of 32 so that higher scores reflect a greater lack of emotional support. Because of the skewed data the scores were recoded into no‐low (scores 0–9), medium (scores 10–13), and high lack of emotional support (scores 14–32).

#### Analyses

Differences in characteristics between the comparison and PTE group were examined using chi‐square tests (for nominal and ordinal variables) and *t*‐tests (continuous variable).

The recovery rate of pre‐existing ADS between T1 and T2 is a central variable in the present study. How the recovery rate is calculated might influence the outcomes and conclusions. We therefore limited the analyses not to one single indicator of recovery, but applied five different indicators of recovery. The following indicators for recovery from ADS between T1 and T2 were used: (1) *recovery* between T1 and T2 according to the reliable change index (RCI; as described by Jacobson and Truax^29^; for calculation see Appendix S1, derived from Dingemans and colleagues^30^), (2) *recovery or improvement* between T1 and T2 using the RCI (see Appendix S1 ^30^), (3) the *reduction* of ADS‐symptom severity between T1 and T2 by 50%,^30^, ^31^ (4) the *reduction* of ADS‐symptom severity between T1 and T2 by 25%,^32^ and (5) not having severe *ADS* at T2 according to the cut‐off of ADS for severe ADS.^24^

Although we selected respondents with severe pre‐existing ADS levels, it is possible that the PTE and comparison group still differed in pre‐existing high ADS levels that may confound results. To rule out this possibility we conducted an ANCOVA with the same control variables, to examine differences in mean ADS scores at T1 between these two groups.

To answer RQ1, differences in the prevalence of recovery according to these five indicators between the PTE and comparison group were examined using multivariate logistic regression analyses with the 11 control variables described above. The five recovery indicators were treated as dependent variables (1 = recovery, 2 = no recovery). Subgroup membership [1 = no PTE (comparison) group, 2 = PTE group] was entered as predictor.

Similar multivariate logistic regression analyses among the PTE group were conducted to answer RQ2, with recovery from severe ADS between T1 and T2 according to the five indicators as predictor, and probable PTSD at T2 as dependent variable. RQ2 was aimed at differences between subgroups within the PTE group. Time passed since the (most drastic) event and experienced stress during the event may affect post‐event mental health problems. We therefore first conducted chi‐square tests to examine if these subgroups differed in time passed since the event and experienced stress during the event. In case they did differ, the variables were added to the list of control variables in the multivariate logistic regression analyses.

According to the DSM5 criteria of PTSD,^21^ symptoms have to last for more than 1 month. For RQ2 we therefore excluded respondents (*n* = 26) who were affected by the (most drastic) PTE less than 1 month before T2.

Control analyses showed that multicollinearity between predictors was not present.

The highest Variance inflation factor (VIF) was 1.075, where the intercorrelation (*R*'s) were based on Kendall's tau‐b (VIF = 1/(1 − *R* ^2^)). The statistical analyses were conducted with IBM SPSS version 28.

## Results

### Characteristics PTE and comparison group

The characteristics of the PTE and comparison groups are presented in Table 1. It shows that both groups mostly share the same characteristics. The three exceptions were that PTE exposed participants significantly more often experienced stressful life‐events between T1 and T2, pre‐existing lack of emotional support at T1, and high PTSD symptom levels at T1 following PTEs in 12 months before T1 than the comparison group.

**Table 1:** Characteristics comparison and potentially traumatic events (PTE) group

	Severe anxiety and depression symptoms at T1	
	Comparison group	PTE group
	(*n* = 325)	(*n* = 162)
	*n* (%)	*n* (%)
Sex		
Males	124 (38.2)	56 (34.6)^ns.^
Females	201 (61.8)	106 (65.4)
Education level at T1		
Low	93 (28.6)	50 (30.9) ^ns.^
Medium	123 (37.8)	64 (39.5)
High	109 (33.5)	48 (29.6)
Primary occupation at T1		
Employed	138 (42.5)	58 (35.8) ^ns.^
Not employed	187 (57.5)	104 (64.2)
Marital status at T1		
Married	119 (36.6)	53 (32.7) ^ns.^
Unmarried	206 (63.4)	109 (67.3)
Financial problems at T1		
No	243 (74.8)	108 (66.7)
Yes	82 (25.2)	54 (33.3)
Stressful life‐events between T1 and T2^†^		
No	191 (58.8)	59 (36.4)***
Yes	134 (41.2)	103 (63.6)
Lack emotional support at T1		
High	76 (23.4)	47 (29.0)*
Medium	77 (23.7)	23 (14.2)
No/low	172 (52.9)	92 (56.8)
High PTSD symptom levels at T1 following PTEs in 12 months before T1		
No	299 (92.0)	122 (75.3)***
Yes	26 (8.0)	40 (24.7)
Use professional help for mental health problems at T1		
No	213 (65.5)	104 (64.2)^ns.^
Yes	112 (34.5)	58 (35.8)
	M (SD)	M (SD)
Age at T1	45.9 (18.2)	52.2 (17.5) ^ns.^

*Note*: *P*‐values *χ* ^2^ and *t*‐test: ^ns.^ = not significant, *P < 0.05, ***P < 0.001.

Abbreviation: PTSD, posttraumatic stress disorder.

^†^ Non‐PTEs varying from burglary, death of a significant other to severe mental health problems of significant other.

Of the PTE group, 24.7% (*n* = 40) were exposed to serious threats, 52.5% (*n* = 85) to accidents, and 22.8.% (*n* = 37) to physical violence between T1 and T2 (most drastic event). More than 80% (83.3%, *n* = 135) experienced moderate to extreme stress during the event.

### Recovery from severe ADS between T1 and T2 among PTE and comparison group (RQ1)

ANCOVA showed that the (adjusted) ADS mean scores at T1 of the PTE and comparison group did not differ significantly (*M* ^comparison^ = 34.96, *M* ^PTE^ = 34.33, *P* = 0.514).

Table 2 shows that, regardless of which indicator for recovery was used, the PTE group significantly less often recovered from pre‐existing severe ADS than the non‐exposed comparison group. The Odd Ratios (OR) and OR adjusted (aOR) for the effects of the control variables were more or less similar across the five indicators for recovery. In addition, according to the first four indicators, only a minority of the PTE and comparison group recovered.

**Table 2:** Recovery from severe anxiety and depression symptomatology

		Recovery from anxiety and depression symptomatology among those with severe symptoms at T1		
Indicators recovery	*n* ^ADS T1^	*n* (%)	OR (95% CI)	aOR (95% CI)
Recovery at T2 according to RCI				
Comparison group^†^	325	106 (32.6)	1	1
PTE group	162	32 (19.8)	0.51 (0.32–0.80)**	0.49 (0.30–0.79)**
Recovery or improvement at T2 according to RCI				
Comparison group^†^	325	120 (36.9)	1	1
PTE group	162	36 (22.2)	0.49 (0.32–0.75)**	0.46 (0.29–0.73)***
Symptom reduction at T2 of 25%				
Comparison group^†^	325	141 (43.4)	1	1
PTE group	162	45 (27.8)	0.50 (0.33–0.75)***	0.51 (0.33–0.79)**
Symptom reduction at T2 of 50%				
Comparison group^†^	325	54 (16.6)	1	1
PTE group	162	12 (7.4)	0.40 (0.21–0.77)**	0.40 (0.20–0.80)**
Recovery at T2 according to‐cut off (score >44)				
Comparison group^†^	325	187 (57.5)	1	1
PTE group	162	73 (45.1)	0.61 (0.41–0.88)**	0.66 (0.44–0.998)*

Abbreviations: 95% CI, 95% confidence interval; ADS, anxiety and depression symptoms; aOR, OR adjusted for gender, age, education level, marital status, employment status, financial problems, lack of emotional support, high posttraumatic stress disorder (PTSD) symptom levels, and use of mental health services at T1, stressful life‐events between T1 and T2, time survey (during pandemic or not); OR, Odds ratio; RCI, Reliable Change Index; PTE, potentially traumatic events.

^†^ Reference category.

* *P* < 0.05;

** *P* < 0.01;

*** *P* < 0.001.

From this table can be derived that, dependent on the criterion of recovery, between 54.9% (“score >44”criterion) and 92.6% (“50% reduction”‐criterion) of the PTE group with severe ADS at T1, did *not* recover from severe ADS between T1 and T2.

### Associations between recovery ADS between T1 and T2, and probable PTSD at T2 among the PTE group (RQ2)

Control‐analyses showed that the non‐recovery groups significantly more often reported quite a bit or extreme stress during the event than the recovery groups according to two of the five recovery indicators, but did not differ in time passed since the event. We therefore added stress during the event to the list of control variables in the regression analyses.

The associations between recovery from ADS according to the five indicators on the one hand and probable PTSD at T2 on the other hand, are presented in Table 3. They show that, regardless of the recovery criterion, the respondents in the PTE group who *did not* recover significantly more often suffered from probable PTSD at T2 compared to the respondents in the PTE group who *did* recover from pre‐existing ADS. The large majority of the non‐recovery subgroups (64.8%–86.8%), in sharp contrast to the recovery subgroups (0%–25.0%), had probable PTSD at T2 and thus typically suffered from comorbid severe ADS and probable PTSD.

**Table 3:** Associations between recovery and probable posttraumatic stress disorder (PTSD) among potentially traumatic events (PTE) group

		Probable PTSD at T2		
Indicators recovery	*n* ^ADS at T1^	*n* (%)	OR (95% CI)	aOR (95% CI)
Recovery at T2 according to RCI				
Yes^†^	26	6 (23.1)	1	1
No	110	75 (68.2)	7.14 (2.64–19.35)***	8.96 (2.55–31.48)***
Recovery or improvement at T2 according to RCI				
Yes^†^	30	7 (23.3)	1	1
No	106	74 (69.8)	7.60 (2.96–19.49)***	10.93 (3.38–35.36)***
Symptom reduction at T2 of 25%				
Yes^†^	39	9 (23.1)	1	1
No	97	72 (74.2)	9.60 (4.01–22.98)***	12.57 (4.06–38.90)***
Symptom reduction at T2 of 50%				
Yes^†^	11	0 (0.0)		
No	125	81 (64.8)	nc.	nc.
Recovery at T2 according to‐cut off (score >44)				
Yes^†^	60	15 (25.0)	1	1
No	76	66 (86.8)	19.80 (8.17–47.99)***	26.33 (8.21–84.99)***

ADS, anxiety and depression symptoms; aOR, OR adjusted for gender, age, education level, marital status, employment status, financial problems and lack of emotional support, high PTSD symptom levels, and use of mental health services at T1, stressful life‐events between T1 and T2, time survey (during pandemic or not), stress during event. 95% CI = 95% confidence interval. nc. = not computable because prevalence in (small) reference category is zero; OR, odds ratio; RCI, Reliable Change Index. Because of the 1‐month criterion of PTSD, the numbers are lower than in Tables 1 and 2

^†^ Reference category.

*** *P* < 0.001.

In order to exclude the possibility that the presence of ADS at T2 and not the lack of recovery is responsible for the last findings, we compared the prevalence of probable PTSD at T2 among by PTEs affected respondents with severe ADS at both T1 and at T2 (who did not recover according cut‐off of ADS for severe AD) with the prevalence of by PTEs affected respondents with severe ADS at T2 but not at T1 (*n* = 70). For this purpose, similar multivariate logistic regression analyses were conducted with probable PTSD as dependent variable and, besides group membership (1 = ADS at T2 but not at T1, 2 = not recovered according to cut‐off of ADS for severe ADS,^24^) the same control variables as predictors. Results showed that by PTE affected respondents with severe ADS at T1 and at T2 significantly more often suffered from probable PTSD at T2 than by PTE affected respondents with severe ADS at T2 but not at T1 (86.6% vs. 52.5%; OR = 5.89, 95% CI = 2.61–13.29, *P* < 0.001; adjusted OR = 6.35, 95% CI = 2.26–17.89, *P* < 0.001).

## Discussion

The aim of the present two‐wave prospective comparative population‐based study was to examine the extent to which recent potentially traumatic events (PTEs) affect the recovery from pre‐existing severe anxiety and depression symptomatology (ADS) and whether the non‐recovery increases the risk of probable PTSD. For this purpose, we applied five indicators of recovery, including among others, the reliable change index (RCI).

Results convincingly showed that, according to the five applied indicators, PTEs during the past year negatively affect the recovery from pre‐existing severe ADS (RQ1), while controlling for relevant potential confounders. Recovery or improvement of ADS according to the RCI, as well as a 25% or 50% reduction of symptom‐scores was about 1.5 as high among the comparison group that was not exposed to PTE in the study period than among the PTE group. In addition, results revealed that non‐recovery is associated with a high risk of probable PTSD compared to the exposed adults who did recover according to the five indicators (RQ2): the large majority of non‐recovered adults in the PTE group had probable PTSD (64.8%–86.8%) in contrast to their recovered counterparts of whom a (small) minority had probable PTSD at T2 (0%–25.0%) according to the five indicators. It should be noted that especially the number of by PTE affected respondents who recovered according to the RCI indicator (*n* = 26) and according to Recovery or improvement indicator of recovery (*n* = 30) was small. However, given the very large differences in the prevalence of probable PTSD we consider it not very likely that larger samples would substantially or meaningfully change the observed patterns. Importantly, the use of mental health services did not differ significantly between the PTE and comparison group (35.8% and 34.5% respectively) and we controlled for services use in the analyses. The prevalence of services use among those with mental health problems (about a one third used services) is in line with the Dutch NEMESIS studies on 12‐month use of services (about one third) among those with mental disorder.^33^, ^34^, ^35^

To the best of our knowledge, to date there are no prospective studies on recent PTEs among adults to compare these findings with. Nevertheless, the strong co‐occurrence between probable PTSD and post‐event severe ADS seems in line with the meta‐analysis of Rytwinski and colleagues^36^ on the co‐occurrence between PTSD and major depression disorder.

The results also showed another important pattern. Across the five criteria, the mean number of respondents with pre‐existing ADS that recovered was 40 ((32 + 36 + 45 + 12 + 73)/5) and the mean number that did *not* recover was 122 (162–40). This suggests that more than three‐quarters of adults exposed to recent PTEs with post‐event severe ADS may be viewed as adults who did not recover from pre‐existing ADS 1 year earlier, and thus suffered from persistent or chronic severe ADS. Although effective therapies are available for the treatment of PTSD, the dropout rates of PTSD‐patients with or without comorbid depression range widely^37^, ^38^ and comorbid depression might inhibit optimal response in treatments for PTSD.^39^, ^40^ Our finding that post‐event severe ADS are often pre‐existing (c.q. persistent or chronic) in nature, may help to explain why PTSD‐patients dropout of treatments or do not show improvement and why comorbid depression negatively affects treatment outcomes.^41^, ^42^

### Strengths and limitations

Major strengths of the present study are the prospective comparative study design, use of a longitudinal panel based on a large traditional probability sample of the Dutch population, multivariate statistical analyses controlling for potential confounders such as pre‐existing lack of emotional support, financial problems and exposure to stressful live‐events in the study period, and use of five different indicators for the recovery from severe ADS including RCI. Although we applied validated cut‐off scores for severe anxiety and depression symptomatology and probable PTSD, we did not conduct clinical interviews that would have enhanced our study. As we analyzed data from one post‐event time point only, we cannot quantify the effects of PTE on ADS recovery in terms of additional time needed for recovery. In addition, given the cell counts we were not able to examine the effects of different types of PTEs, c.q., whether recent serious threats, accidents, or physical violence more often hinder or delay recovery. We examined the effects of more recent or fewer single PTEs among adults and future studies are warranted to examine the extent to which our findings are applicable to continuous PTEs (Type II trauma^43^) such as childhood abuse and war.

In addition, we were not able to examine possible differences in the recovery from pre‐existing ADS and the risk of probable PTSD between other relevant subgroups such as different age categories, males and females, and those with and without financial problems. The time between T1 and T2 was 1 year, and we used five indicators of recovery based on the T2 data. We cannot rule out the possibility that some by PTE's affected respondents who did not recover according to the T2 data, may have been (almost) symptom free during a short time between T1 and the PTE. In these cases, PTE may still be viewed as events that hinder recovery in the sense that they obstruct a stable recovery.

PTSD, anxiety and depression symptomatology cover important post‐event mental health problems. However, the possible effects of PTEs on mental health may also include other mental health problems such as substance abuse,^44^ and sleep problems.^45^ Future prospective comparative studies among adults are warranted to examine the extent to which recent PTEs hinder the recovery from other pre‐existing mental health problems, and the relationships of (non‐) recovery from these pre‐existing mental health problems with probable PTSD.

In the present study we examined the effects of PTEs on the recovery from severe pre‐existing anxiety and depression symptoms (MHI‐5 sum scores of anxiety‐ and depression‐related items). We were not able to examine the extent to which the effects of PTEs on the recovery from pre‐existing severe anxiety symptoms differs from the effects of PTEs on the recovery from pre‐existing severe depression symptoms (and thereby on development of PTSD). Future prospective studies are needed to test if they differ. Given the strong associations between anxiety and depression symptoms^46^ our hypothesis would be that the effects of PTEs on the recovery from severe pre‐existing anxiety symptoms hardly differs from the effects of PTEs on the recovery from severe pre‐existing depression symptoms.

### Final conclusions

Despite these limitations, the finding that recent PTEs affect the recovery from severe ADS substantially and that a lack of recovery increases the risk of probable PTSD strongly suggests that health care professionals, including general practitioners and occupational physicians, should not exclusively explore the current potentially traumatic experience and its immediate consequences, but also carefully screen for pre‐existing ADS when by PTE affected suffer from post‐event severe ADS. On the one hand, increased efforts of monitoring (“watchful waiting”^47^) may be indicated due to the elevated risk of PTSD development in this subgroup. Findings suggest that victim support professionals are often in the unique position shortly after PTEs to ask people about pre‐existing ADS. Of course, involved professionals should be aware of the risk of recall‐bias of by PTEs affected individuals when retrospectively assessing pre‐existing severe ADS.^48^, ^49^ Information obtained from relatives about pre‐existing ADS and possible treatment histories or medical records may, if possible or accessible, help to gain further insight in pre‐existing ADS. On the other hand, persistent mental health conditions may require different treatment approaches as compared to current mental health impairments in the aftermath of recent PTE,^50^ as chronic and complex problems are more difficult to treat.^51^

## Disclosure statement

The authors declared no potential conflicts of interest concerning this article's research, authorship, and/or publication.

## Author contributions

P.V., C.C., and L.W. were involved in the conception and design of the study, P.V. and M.D. in the acquisition, and P.V., L.L. and L.W. in the analysis of data. All authors contributed to drafting the manuscript.

## Supporting information


**Appendix S1.** Computation RCI.
