# Supplementary material for: The effects of potentially traumatic events on the recovery from pre‐existing anxiety and depression symptomatology and the risk of PTSD
Source: Psychiatry Clin Neurosci. 2024 Aug 20;78(11):696–702. doi: 10.1111/pcn.13725 (PMC11804911; doi:10.1111/pcn.13725)
Supplement: Supplementary file 1 — Appendix S1. Computation RCI. [file PCN-78-696-s001.docx]

**Appendix 1 Computation RCI**

As described, lower ADS scores indicate higher ADS levels. To apply the RCI we recoded the original ADS scores so that higher scores indicate higher ADS levels (100 minus original ADS-score). The calculation of the number of respondents who recovered/improved according to the 25% and 50% reduction were also based on the recoded scores.

1.) RCI = 1.96*√ 2 (SEM)^2^, with SEM = SD^general population^*(1-Cronbach’s alpha^general population^); and

2.) CO = ((M^severe ADS^*SD^general population^) + (M^general population^*SD^severe ADS^))/ (SD^general population^ *SD^severe ADS^).

Recovery (of ADS) = ((S^difference T1-T2^ ≥ RCI) and (S^T1^ ≥ S^T2^) and (S^T2^ < CO))

Improvement (of ADS = ((S^difference T1-T2^ ≥ RCI) and (S^T1^ ≥ S^T2^) and (S^T2^ ≥ CO))

Other = group without recovery and improvement subgroups.

S^T1 =^ Score^T1^ (individual ADS scores of those with severe ADS at T1)

S^T2 =^ Score^T2^ (individual ADS scores of those with severe ADS at T2)

S^difference T1-T2 =^ Score^T1^- Score^T2^
